# Square-edge intraocular lenses and epithelial lens cell proliferation: implications on posterior capsule opacification in an in vitro model

**DOI:** 10.1186/1471-2415-15-5

**Published:** 2015-01-19

**Authors:** Rita Mencucci, Eleonora Favuzza, Carlotta Boccalini, Jean-Jacques Gicquel, Laura Raimondi

**Affiliations:** Department of Surgery and Translational Medicine – Eye Clinic, University of Florence, Florence, Italy; Department of Ophthalmology, Poitiers University Hospital, Poitiers, Cedex, France; Department of NEUROFARBA, section of Pharmacology, University of Florence, Florence, Italy

**Keywords:** Posterior capsule opacification, Intraocular lens, Square edge, Cataract

## Abstract

**Background:**

To evaluate lens epithelial cell (LEC) proliferation with two different designs (one-piece or three-piece) of hydrophobic acrylic IOLs with 360° square optic edge using an in vitro culture model of posterior capsule opacification (PCO).

**Methods:**

This experimental study was conducted at the Department of NEUROFARBA, Section of Pharmacology, University of Florence, Italy. Human LECs were seeded and cultured in transwell cell culture inserts coated with a type-IV collagen membrane on which an IOL (one-piece Tecnis-1 or three-piece AR40E, Abbott Medical Optics Inc.) had been previously placed. As control, cells were plated on the insert membrane without an IOL. At day six (cells confluent in controls) IOLs were removed and cell counting, viability and cell density under and outside the IOLs were evaluated.

**Results:**

No statistically significant difference in the number of cells (p > 0.05) between inserts with the one-piece and three-piece IOLs was found. Cell density in the area under each IOL was significantly lower than in the area outside of it (p < 0.05), or in the control insert. (p < 0.05). Cell density under the single-piece IOL was not significantly different from that under the three-piece IOL (p > 0.05).

**Conclusions:**

A 360° sharp-edge played a crucial role in avoiding LEC migration under the IOL and preventing the formation of PCO after cataract surgery. Long term clinical evaluation is necessary to estimate functional results.

## Background

The proliferation and migration of residual lens epithelial cells (LECs) from the peripheral posterior capsular bag into the space between the capsule and the optic of the intraocular ocular lens (IOL) still remains a postoperative complication of modern cataract surgery. This phenomenon leads to anterior (ACO) and posterior capsule opacification (PCO) and decreased visual acuity [1, 2]. Mechanical [3], pharmacological [4], immunological [5] and surgical factors [6] seem to be related to the presence of PCO after cataract surgery. Advances in intraocular lens (IOL) materials and design attempt to control some of these factors and to avoid PCO development as much as possible. Hollick and colleagues [7] demonstrated several years ago that the presence of LECs on the posterior capsule after cataract surgery was considerably lower with polyacrylic IOLs than with poly(methyl methacrylate) (PMMA) or silicon IOLs. Indeed, in vitro studies have shown that the migration of human LECs under an acrylic IOL is reduced in comparison to PMMA and silicone IOLs [8]. Regarding IOL design, a sharp optic edge has been demonstrated to prevent migration of LEC between the capsular bag and the IOL [8, 9].

In spite of the slowing down or blocking of PCO development with square optic edges, there is still a relevant clinical evidence of PCO [8, 9] and consequently a considerable interest in developing in vitro models allowing the clinician to understand the mechanisms of LEC proliferation. Several in vitro models have been described, mainly based on lens capsular bag systems (organ models) [10–14]: although these models reproduce the human capsular bag-lens environment in the most accurate way, they are quite complex to carry out and the LEC proliferation and migration measurements can be scarcely reproducible due to the high interindividual variability of the ex vivo lens capsule characteristics [15]. On the contrary, in vitro cell culture models [15–18] can be more easily set up.

Animal studies [19] post-mortem analysis [20] and clinical evidence [21] showed that the square-edge interruption at the optic-haptic junction typical of most of the one-piece acrylic IOLs (the so-called Achilles’ heel of these IOLs) [22]can increase the incidence and the severity of PCO in comparison with a continuous posterior square-edge. Recently, a hydrophobic acrylic one piece IOL with a 360-degree square edge (Tecnis-1 ZCB00, Abbott Medical Optics Inc, USA) was introduced in Europe, and some authors found a lower incidence of PCO in eyes implanted with these IOLs compared with interrupted square-edge one-piece IOLs [2]. The aim of our study was to evaluate LEC proliferation and migration with a 360-degree square-edge one-piece IOL (Tecnis-1 ZCB00 from AMO) and a square-edge three-piece IOL (AR40E from AMO) using an in vitro cell culture model.

## Methods

The experiments were performed following the tenets of the Declaration of Helsinki.

### Cell culture

In this study, human LECs were used (B-3 cell line), which were obtained from the American Type Culture Collection (ATCC, Manassas, VA, USA). The cells were maintained in culture medium consisting of Dulbecco’s modified Eagle’s medium (DMEM, Sigma-Aldrich, St. Louis, MO, USA), 20% fetal bovine serum (FBS, Sigma-Aldrich, St. Louis, MO, USA), 100 U/mL penicillin G, 100 μg/mL streptomycin (Sigma-Aldrich, St. Louis, MO, USA), sodium pyruvate (1:50), L-glutamine (1:100), in a humidified atmosphere of 5% CO_2_ at 37°C.

### Intraocular lenses (IOLs)

The two types of IOLs (Tecnis ZCB00 and AR40E from AMO) evaluated in the current study are made of the same hydrophobic acrylic material, have a 6-mm biconvex optic, an overall diameter of 13 mm and a continuous 360-degree posterior square edge to prevent PCO. The Tecnis ZCB00 is a one-piece IOL with aspheric anterior surface and offset haptics, made of the same material as the optic, whereas the AR40E is a three-piece IOL with PMMA angulated haptics (5-degree angle). Both IOL models are produced via lathe-cutting by the same manufacturer. For the purpose of the study, 10 IOLs of the same optical power (+20 Diopters) were selected for each type of design.

### PCO in vitro model

The PCO model previously described by Gotoh et al. [16] was used for our experimental research. Each IOL (n = 20) was placed on a cell culture insert coated with 5 μg/cm^2^ type IV collagen (BD Biosciences, San Jose, CA) in a 12-well culture plate to simulate the formation of PCO after cataract surgery with the implantation of an IOL.

A tiny aluminium weight (0.85 g) [16] was placed on the IOL to maintain contact between the IOL and the collagen membrane. Cells (10^4^) were seeded into each culture transwell and incubated in 700 μl 20% FBS/DMEM. Collagen membrane inserts without IOL (n = 10) were considered as controls.

### Proliferation assay: cell counting and viability

After 6 days (when the cells incubated without IOL reached confluence), the cells present in the whole insert were detached by trypsinization and counted with the trypan blue exclusion test. Cell viability was evaluated throughout the oxidation by the mitochondrial chain of 3-[4,5-dimethylthiazol-2yl]-2,5-diphenyltetrazolium bromide (MTT) to insoluble formazan. The formazan production after 2 h was evaluated colorimetrically at 590 nm, in terms of optical density (OD) [23].

### Proliferation assay: cell density

At day 6 (when the cells incubated without IOL reached confluence), cells were fixed with 100% methanol and stained with hematoxylin-eosin. Cells present in six independent zones (under and outside the IOL) were photographed and counted using an inverted-phase microscope (TMS, Nikon Instruments Europe BV, Netherlands).

**Figure 1:**
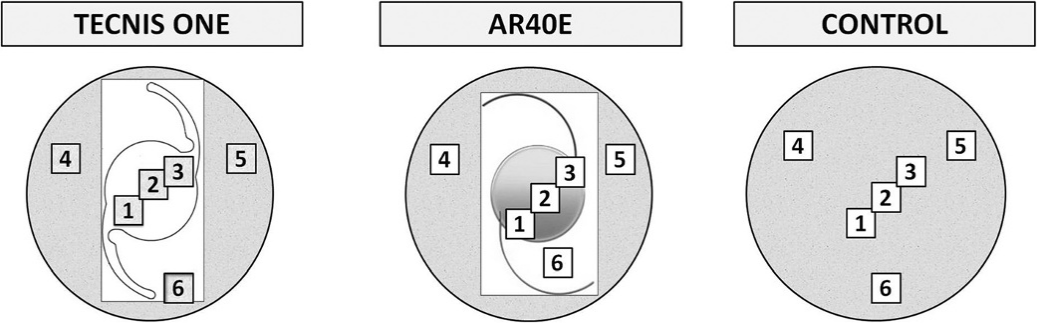
**Diagram showing the experimental procedure for the evaluation of the cell density in the proliferation assay.**

Mean values from cell counting were determined using a 21 mm^2^ grid (Figure 1).

### Statistical analysis

Calculations were made using GraphPad Prism and Instat version 4.0 for Windows (GraphPad Software, San Diego, CA, USA) was used for statistical analysis. Results for each IOL and the control model were the mean ± SD of at least 5 experiments run in duplicate. The Wilcoxon Rank Sum test was applied to assess the comparisons between cell counting and density for each IOL and the control model, using in all cases the same level of significance (p < 0.05).

## Results

### Cell counting and viability

**Figure 2:**
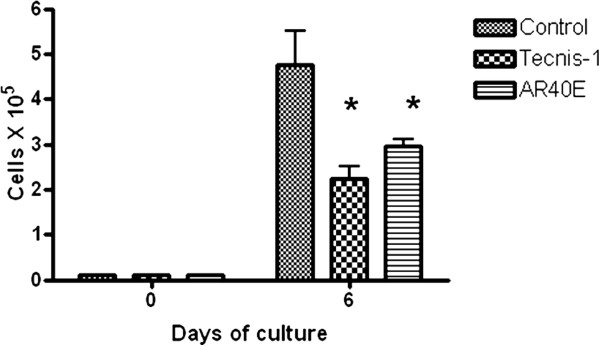
**Cell number counting during the 6-day period of culture.** *p < 0.05 vs Control at day six.

Figure 2 shows the changes over the period of culture in the cell counting for each IOL as well as for the control inserts. As shown, the cell number in inserts containing IOLs was significantly lower (p < 0.05) at day 6 than that in controls. In contrast, no statistically significant difference was found in the number of cells contained in the inserts with the Tecnis ZCB00 and AR40E IOLs (p > 0.05). Regarding cell viability, the optical density at 590 nm after the MTT test was 0.208 ± 0.050 in controls, 0.123 ± 0.010 in Tecnis-1 inserts and 0.116 ± 0.012 in AR40E inserts. All cells present in each insert were viable.

### Cell density

**Figure 3:**
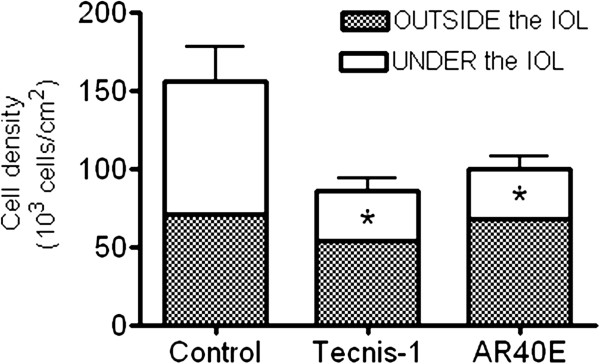
**Cell density outcomes at day six for each IOL as well as for the control inserts, outside and under the IOLs.** *p < 0.05 vs Control at day six.

Figure 3 summarizes the results of the cell density test at day 6 for each IOL, as well as for the control insert. As shown, cell density in the area under each IOL was significantly lower than that found outside of it (p < 0.05, Figure 3). Likewise, cell density was significantly higher in the control insert compared to those containing the IOLs (p < 0.05, Figure 3). Cell density under the Tecnis ZCB00 IOL was not significantly different from that under the AR40E IOL (p > 0.05, Figure 3).

**Figure 4:**
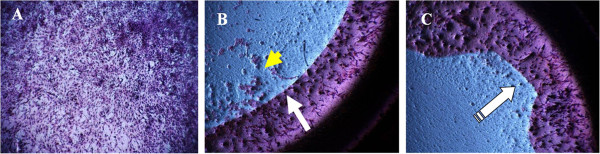
**Lens Epithelial Cells on transwell collagen membrane after IOL removal, cell fixation and hematoxylin-eosin staining (4X). A)** Control: cell confluence at day 6. **B)** AR40E insert: clear separation between cells under the IOL and those beyond the optic square-edge barrier. **C)** Tecnis-1 insert: Cell migration at the optic-haptic junction prevented by the 360° sharp optic edge.

In microscopic images (Figure 4A,B and C, 4X), cells were shown to concentrate at the square-edge barrier (Figure 4B and C), with low migration of cells under the IOL (Figure 4B and C). Furthermore, the analysis of the microscopic photographs revealed the importance of the 360° square edge to avoid cell migration at the optic-haptic junction (Figure 4C).

## Discussion

The present study shows that in our experimental conditions a 360° sharp optic edge acts as an effective barrier to LEC migration.

Similarly, Kurosaka and colleagues [15] found in another in vitro study that an acrylic sharp-edge IOL and a PMMA IOL with similar edge design inhibited LEC migration more effectively than silicone and PMMA rounded-edge IOLs. This is consistent with the clinical evidence reported [2, 8, 9]. Specifically, Nixon and Woodcock found in a comparative study between eyes implanted with continuous-edge and interrupted edge IOLs that the mean EPCO (software for the Evaluation of Posterior Capsule Opacification) score was significantly higher for eyes with the interrupted-edge IOL compared to the 360°square-edge (0.39 vs. 0.08, p = 0.012) [2]. On the contrary, other authors showed similar incidence of PCO in eyes implanted with interrupted square-edge IOLs, in comparison with 360° square-edge IOLs [24]. Anyway, even if this concept is still under debate, a sharp uninterrupted optic edge can be considered an important factor in preventing PCO formation.

In the last years the researchers’ interest towards in vitro PCO models that allow the study of PCO cellular mechanisms and potential prevention factors is rising. Human lens epithelial cell behaviour in different experimental conditions, with media containing different protein or growth factor concentrations has indeed been deeply studied [10]. The capsular bag organ models use animal or human lens capsule, isolated from donor eyes after sham cataract surgery with or without an intraocular lens implantation [10–14]. Various methods have been described in order to support the capsular bag during culture: external fixation of the capsule to the culture dish using glue [13] or entomological pins [10, 14], the fixation of the ciliary body [11], or internal capsule expansion using Capsular Tension Rings (CTRs) [12]. The main limitations of these models are the potential obscuration of the view of the equatorial area and the interference with LEC migration (CTRs models), a possible distortion of the capsular shape, the forced apposition of the posterior and the anterior capsule and potential capsule tears formation (“pin models”) [11]. Moreover, LECs from individuals of different ages and characteristics can have different growth pattern and behaviour, affecting the reproducibility of the model [15].

In vitro cell culture models, on the other hand, try to reproduce the capsular bag system using transwell inserts coated with bovine [15], Type I or Type IV collagen membrane [16, 17], or PMMA Petri dish [18], on which an IOL can be placed and LECs (immortalized cell lines or primary/secondary cultures) can be seeded and cultured. These materials are commercially available and are easy to handle.

They have been successfully used in previous studies evaluating different aspects of PCO formation [15–17]. Kurosaka et al. [15] studied how acrylic and others IOLs influence LEC migration, concluding that acrylic IOLs inhibited LEC migration by not only a sharp edge but also other factors, such as adhesive properties of the material. Okajima and coauthors [17] evaluated the effect of coating a hydrophobic acrylic IOL with poly(2-methacryloyloxyethyl phosphorylcholine) (MPC) on LEC migration, showing that this coating suppressed adhesion and proliferation of LECs.

We used the in vitro model described by Gotoh and colleagues [16]. This model was originally developed to examine the effect of matricellular protein SPARC (secreted protein, acidic and rich in cysteine) and transforming growth factor (TGF)-β on PCO [16].

One of the most important parameters that can affect the evaluation of LEC behaviour and growth pattern in in vitro culture models in the presence of different IOLs, is the difficulty in reproducing the forces that act in vivo on the IOL keeping it in contact with the posterior capsule. Tetz and Wildeck [18] tested and compared different weights of various materials on IOLs placed on a culture dish, concluding that a silver weight of 0.7 g was able to fix the IOL without blocking cell growth under it and causing no cell reaction. Gotoh et al. [16] used instead an aluminium weight of 0.85 g, that in our experimental condition was able to give stability to the IOL without causing cell death or reaction.

Anyway also in the organ models the apposition between the anterior and the posterior capsule is a crucial factor and can vary between the different models [11].

The main advantage of in vitro cell culture models is their acceptable reproducibility in the same experimental conditions. The Gotoh model allowed us to compare easily the LEC behaviour in presence of IOLs with different designs.

Nevertheless, it should be considered that this model, such as all the available in vitro models of PCO, is not able to fully reproduce the complex environment of the capsular bag-lens system. Moreover, the influence of the anterior capsule and haptic angulation on PCO are not taken into account.

In our study we found a significant difference between the number of cells present in the area outside the IOLs and under the IOLs, suggesting a barrier effect of the posterior sharp edge of the optic of both IOL types. In the transwells with the Tecnis-1 IOL, the cell migration under the optic-haptic junction, generally considered a weak point of the edge barrier of the single-piece IOLs, was minimized by the peculiar design that guarantees a continuous posterior sharp-edge. This offset haptic design allows for a 360° sharp optic edge preventing LEC migration. Additionally cell migration is prevented by the 3 point fixation (TriFix design) which pushes the lens against the posterior capsular bag and keeps it in a secure position.

Clinical studies [2] showed not only a low rate of PCO but also a relatively good centration and rotational stability of this 1-piece design, which again is supported by the 3-point fixation that allows a sustained contact between the IOL and the posterior capsule [25].

## Conclusions

In conclusion, our experimental results suggest that a 360° IOL sharp optic edge is a crucial factor to avoid LEC migration and therefore the formation of PCO after cataract surgery. Furthermore, a similar behaviour in inhibiting LEC migration is present in a single-piece and a three-piece IOL with 360-degree optic edge design and comparable material properties. Long term clinical evaluation is necessary to investigate functional results, development of PCO and the influence of the anterior capsule and of the haptic angulation in these two IOL models.
